# Semantics Aware Dynamic SLAM Based on 3D MODT

**DOI:** 10.3390/s21196355

**Published:** 2021-09-23

**Authors:** Muhammad Sualeh, Gon-Woo Kim

**Affiliations:** Intelligent Robotics Laboratory, Control and Robotics Engineering Department, Chungbuk National University, Cheongju 28644, Chungbuk, Korea; er.sualeh@gmail.com

**Keywords:** semantics, 3D multiple object detection, multiple object tracking, dynamic SLAM

## Abstract

The idea of SLAM (Simultaneous Localization and Mapping) being a solved problem revolves around the static world assumption, even though autonomous systems are gaining environmental perception capabilities by exploiting the advances in computer vision and data-driven approaches. The computational demands and time complexities remain the main impediment in the effective fusion of the paradigms. In this paper, a framework to solve the dynamic SLAM problem is proposed. The dynamic regions of the scene are handled by making use of Visual-LiDAR based MODT (Multiple Object Detection and Tracking). Furthermore, minimal computational demands and real-time performance are ensured. The framework is tested on the KITTI Datasets and evaluated against the publicly available evaluation tools for a fair comparison with state-of-the-art SLAM algorithms. The results suggest that the proposed dynamic SLAM framework can perform in real-time with budgeted computational resources. In addition, the fused MODT provides rich semantic information that can be readily integrated into SLAM.

## 1. Introduction

In the realm of robotics, the SLAM paradigm is a well-established research area. Even though there are several efficient solutions to the problem, most of the approaches rely on the static world assumption [1]. The use of current algorithms in a real-world setting, where a dynamic and unstructured environment is a given, is hampered by this assumption. Although the advances in data driven approaches have enabled near-real-time environmental perception, and have shown promising performances [2,3], real-time requirements become a hurdle in resource-constrained computing platforms. Incorporating the semantics information from the environment into a geometric SLAM formulation also necessitates a 3D-pose of the detected objects, as well as semantic segmentation [4,5,6], which further adds to the complexity [4,5,6]. The learning-based techniques inherently necessitate bulky computing resources to meet the real-time requirements. The SLAM techniques that utilize such approaches require a lot of computing power or cannot guarantee real-time operation.

The objective of this work is to effectively merge the two broad paradigms of SLAM and 3D MODT, such that both complement the individual findings while being capable of performing independently. The feature based visual SLAM approaches rely on feature tracking to estimate the camera pose over time. Unless the region of visual feature is highly dynamic, it is hard to categorize and filter the features pertaining to the dynamic regions. Thus, if left untreated, the information pertaining to dynamic regions of the scene gets incorporated in the pose estimation process, which leads to an inaccurate SLAM system. Traditionally, semantic segmentation masks are provided alongside the image to enable SLAM for an informed selection of visual features at the early stage. This process is however computationally demanding and real-time constraint is often compromised. Furthermore, the semantic segmentation only provides class labels and contour of the detected objects. Hence, there is a need for an alternate approach that can effectively provide equivalent semantic information of the environment without demanding extraordinary computational demands and that can guarantee real-time operation.

The capability of detecting and avoiding information related to the dynamic objects in the environment for mapping and localization purposes is the concept underlying the term dynamic SLAM. The dynamic objects are often considered as outliers, and the outlier rejection techniques such as RANSAC are employed to filter such data. In the literature, several attempts are made to deal with the presence of dynamic objects under feature-based SLAM [7,8,9,10] and direct SLAM approaches [11,12,13,14]. The further categorization of dynamic SLAM is based on the type of semantics integration adopted. The loosely coupled approaches [8,15,16,17,18] perform environmental perception and SLAM separately. On the other hand, tightly coupled approaches solve both problems in an integrated fashion [19,20,21,22,23,24,25]. Both approaches have their pros and cons, but the core issue boils down to the computational resource requirements of perception and real-time capability of the entire framework, which is largely left unattended.

The proposed framework operates on a Visual-LiDAR setup that provides calibrated and time synchronized inputs. The core of the framework is a lightweight neural network model that takes the image sequence from camera and produces classification and localization of objects in the image frame. LiDAR data, on the other hand, and treated by the MODT module, which clusters and tracks the potentially trackable objects. The sensor fusion module utilizes the tracking information to associate visual classifications with tracked clusters. The classified point pertaining to the tracked dynamic objects is projected onto the image frame and up sampled to form a dynamic region mask. The mask of the dynamic region coupled with the image is provided to visual the SLAM module. This enables the SLAM module to make an informed selection of visual features by avoiding the features pertaining to the dynamic regions of the scene.

The key contributions in this work are the semantic mask generation, point cloud classification, visual-LiDAR based MODT, visual features selection in SLAM, and the integration of SLAM and MODT. The mask is generated by fusing the information from the visual detector and LiDAR based tracker. Instead of frame-wise classification of the LiDAR point cloud, the tracked clusters are temporally classified using visual detection information. The SLAM module can make effective use of masks by avoiding the features pertaining to dynamic regions of the scene.

To evaluate and analyze the effect of the proposed dynamic SLAM framework, a comparison is made between basic ORB-SLAM2 [26] and the proposed MODT based dynamic SLAM framework. The Tracking KITTI Datasets [27] are used for fair evaluation against the provided ground truths over well-established evaluation metrics. The results suggest that the proposed approach is an effective solution for the dynamic environments. Furthermore, the framework is evaluated over selected sequences of Raw KITTI datasets [28] are for comparison with state-of-the-art dynamic SLAM approaches.

The remainder of the paper is structured as follows: in Section 2, related works are briefly described. In Section 3, an overview of the framework is presented. The proposed methodology is introduced in Section 4. The evaluation results and comparison with state of the art are laid down in Section 5, followed by conclusions in Section 6.

## 2. Related Works

In recent years, several attempts have been made to address the dynamic SLAM problem by integrating semantics and environmental perception in SLAM. A feature-based SLAM paradigm [7] projects the map features onto the current frame to classify the dynamic part of the scene. Three-dimensional object tracking is performed in [8] to identify dynamic objects in the scene. Similarly, detection and tracking approaches are proposed in [9] but are restricted to humans being the dynamic objects. The technique proposed in [10] assigns probabilistic weights to the objects, based on the class. The direct SLAM approaches for dynamic environments have also been proposed [11,12,13,14]. The stereo camera-based scene flow representation is utilized in [11] to detect moving objects. RGB optical flow-based segmentation is performed in [12]. A method is proposed in [13], that exploits consecutive depth images to differentiate static and dynamic parts of the scene. Similarly, the work in [14] focuses on the intensity difference of the consecutive RGB images. The above-mentioned approaches consider the assumption that the dynamic object in the scene remains dynamic for the entire view time. Consequently, the dynamic object at rest is still considered static, which eventually becomes a part of the mapping process. The advances in the deep learning paradigm have enabled prior classification of dynamic objects based on the classes. References such as [8,9] can detect and classify the dynamic objects; however, detection of changes produced in the environment by static objects is still challenging. A fusion of multi-view geometry and deep learning techniques caters to such shortcomings [29,30].

The MODT integrated SLAM paradigm can broadly be classified into the categories of Loosely Coupled and Tightly Coupled approaches [25]. The loosely coupled approaches perform MODT and SLAM separately, whereas tightly coupled approaches operate in an integrated fashion [19,20,21,22,23,24,25].

Conventionally MODT based SLAM approaches are loosely coupled [8,15,16,17,18] and solve MODT and SLAM separately. An implementation in [8] tracks 3D objects and exploits the information provided by the SLAM, and the tracker allows the features pertaining to the objects. A derivation of the Bayes formula of SLAM with tracking of moving objects is devised in [15]. A graph-based approach in [16] makes use of Expectation Maximization, allowing the landmarks to be dynamic. A dense mapping algorithm is proposed in [17] that reconstructs the static background and the dynamic objects in the scene using stereo vision. A dense mesh tracking approach is proposed in [18] that utilizes visual-inertial SLAM in conjunction. The approach is focused on humans, tested on only a simulated environment, and largely relies on camera pose estimation. That is, if the camera pose estimation fails, the MODT also fails. The work in SLAMMOT [31] established a mathematical framework to integrate filtering-based SLAM with dynamic object tracking. Later, an RGB-D camera was employed with the same technique for dense reconstruction of an indoor scene, together with dynamic objects using semantic segmentation [13,14,29,30]. Further, in dense approaches Mask-Fusion [32] and MID-Fusion [33], z techniques were deployed for more accurate semantic segmentation of the dynamic objects in the scene.

Tightly Coupled MODT and SLAM approaches aim to merge information from static and dynamic parts of the scene into a single framework to enhance the estimation accuracy. The work in [19] presented the idea of a tightly coupled approach, however, with limited comparable results. Being end-to-end trained approach estimates 3D pose and dimensions of cars jointly with camera poses. Although the approach provides accurate pose estimations, it suffers with the loss of generality, and thus, huge data would be required to track generic objects. The CubeSLAM [20] is a monocular SLAM based approach that generate 3D bounding box proposals based on 2D bounding boxes and vanishing points. The approach assumes objects to have constant velocity for a fixed time and exploits the constraints pertaining to road structure, non-holonomic wheel motion, and visibility of the cuboid view of objects. The approach in ClusterSLAM [24] proposes a SLAM back end to identify rigid bodies and to estimate the motion. The approach relies on the landmark tracking and association quality. A technique in ClusterVO [21] models the object points for the probability of being dynamic. VDO-SLAM [23] capitalizes on dense optical flow to identify the number of tracked points on the dynamic objects. The bundle adjustment is implemented with cameras, objects, and points; however, at the cost of high computational demand. Similarly, DynaSLAMII [25] proposed a tightly coupled approach for MODT and SLAM that performs the bundle adjustment between camera, points, and objects. The performance relies on high quality semantic segmentation and thus, high computational resources are required.

In contrast to the existing techniques, the proposed framework in this work is unique and hybrid in a way that MODT and SLAM is performed over different sensor modalities. 3D MODT is performed with respect to the vehicle frame of reference and independent of SLAM’s estimated pose, whereas SLAM can take full use of semantic MODT information as and when required for feature tracking, mapping, or loop closures. The advantage of such integration is manifold. The object tracking continues even if the pose estimation of SLAM fails. Furthermore, SLAM can make use of masked dynamic regions and provide pose information to MODT to obtain tracking information in a common frame of reference.

## 3. System Overview

**Figure 1:**
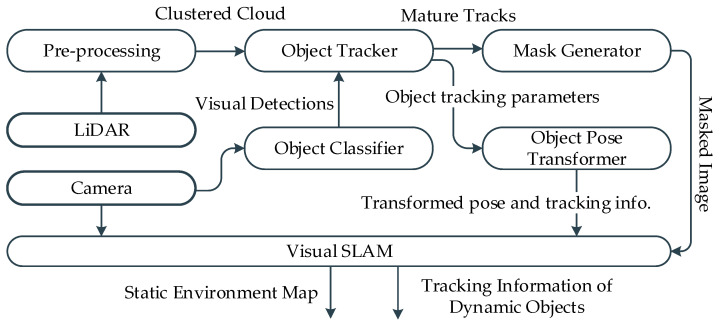
Framework for joint MODT and SLAM.

The system pipeline of the proposed framework assumes that the platform is equipped with visual and LiDAR sensors. The visual sensor is primarily used for SLAM and object detection, whereas the LiDAR sensor is used for spatial object tracking. The framework is built on top of ORB-SLAM2 [26] with an additional stream of input for the masked image of dynamic objects. In parallel, an Interactive Multiple Model–Unscented Kalman Filter–Joint Probabilistic Data Association Filter (IMM–UKF–JPDAF) based tracker is operated to track objects in 3D space. To classify the objects, a lightweight visual detector YOLO-v3 [34] is deployed that operates on a reduced image resolution. This increases the inference speed of the visual detector at the cost of frequent missed detections of small and partially occluded objects. However, this shortcoming is leveraged by the object tracker, which preserves the classification history of the tracked objects. The LiDAR point cloud clusters pertaining to the tracked objects are projected onto the image frame and up sampled to generate the dynamic objects mask. The dynamic object mask is exploited by the visual SLAM to choose the visual features for odometry and/or mapping. Figure 1 shows the block diagram of the framework with individual modules.

## 4. Proposed Framework

**Figure 2:**
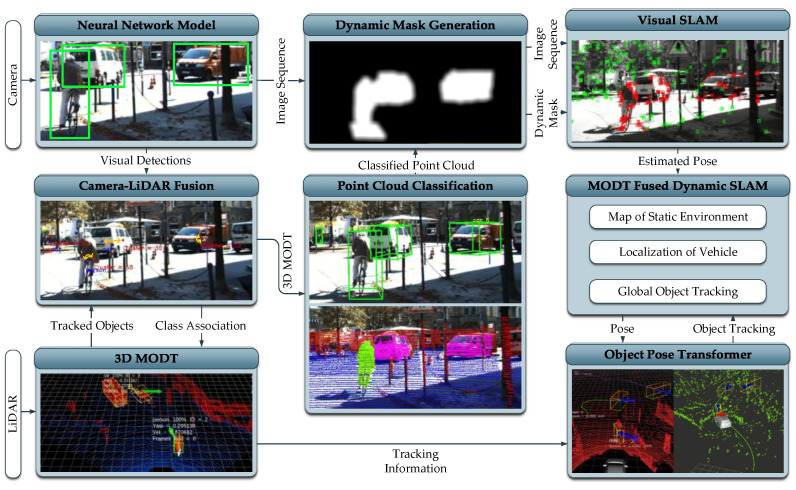
Proposed MODT integrated Dynamic SLAM framework.

The proposed framework is comprised of several modules that interact to solve the dynamic SLAM problem. The operations of individual modules are briefly described in the subsequent subsections and the block diagram is presented in Figure 2. The implementation and evaluation of the framework is based on sensor setup involving stereo camera and 64 channel LiDAR. A stereo camera is used for visual SLAM and visual object detection, whereas a LiDAR sensor is utilized for spatial object detection and tracking. Furthermore, the computational environment used for developing and evaluating the proposed framework is constituted by a desktop computer having an Intel Core i7-7700 CPU with 16 GB of RAM, and Nvidia GTX 1060 GPU. The system runs the Robot Operating System (ROS) “Melodic Morenia” middleware on top of Ubuntu 18.04.5 LTS. The framework is implemented and evaluated in a ROS environment to ensure real-time capability.

### 4.1. 3D MODT

**Figure 3:**
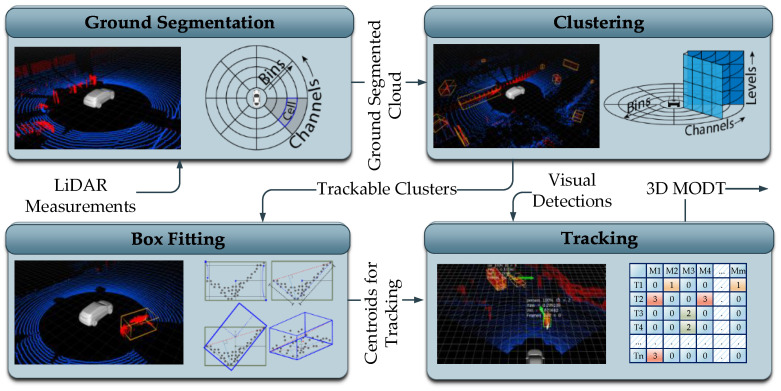
3D MODT module in the proposed framework.

The 3D MODT module preprocesses the LiDAR point cloud and maintains the tracks of potentially trackable objects. The module has four subcomponents: ground segmentation, clustering, box fitting, and tracking. Each subcomponent is briefly discussed in the subsequent subsections and information flow is described in Figure 3. The LiDAR data after getting treated by the submodules of ground segmentation, clustering, and box fitting are presented to the tracking submodule as measurements for tracking. The tracking submodule maintains a temporal record of tracked objects’ dimensions and class associations received from the visual detector. The 3D MODT module provides 3D MODT information with respect to the vehicle reference frame to the camera-LiDAR fusion and pose transformer modules, respectively.

#### 4.1.1. Ground Segmentation

The ground segmentation is a part of the LiDAR preprocessing step, where LiDAR measurements pertaining to the ground are identified and separated from the point cloud for further processing. Several techniques are proposed in the literature to accomplish ground segmentation, with a grid-based approach being the most relevant and effective [35]. The grid-based approach can accommodate the assumptions of the ground being non-planner, and the point-cloud being a merger of multiple LiDARs measurements. Furthermore, the grid-based approach is computationally efficient because of compact data representation. Moreover, the approach can be easily deployed in any arrangement of the sensor setup without requiring training procedures like data driven approaches. The implementation of ground segmentation in this work follows a grid-based approach that considers a non-planar ground.

Initially invalid and out of rage measurements of the LiDAR point cloud are filtered out and the point cloud is converted into a 2D cylindrical representation. The 2D cylindrical representation is composed of channels and bins, as expressed in Figure 3 from the top view. The channels represent the measurements pertaining to the vertical slices of the LiDAR data, whereas bins are the further divisions of the channels based on the distance from the origin. To segment the ground, bins of each channel are traversed to estimate the local ground level, starting from the origin, where the ground level equals the sensor height. The estimated local ground levels together with vertical distribution of the measurements in a bin and absolute slope between consecutive bins are used to set the ground threshold for each bin. The bin-level ground threshold is applied to label the LiDAR measurements as ground and non-ground measurements. The non-ground labeled point cloud is then fed forward for clustering.

#### 4.1.2. Clustering

The clustering of LiDAR point cloud is referred to as the process of grouping the closely existing LiDAR measurements, the approach adopted in this work falls under the hierarchy-based technique [36]. The 2D cylindrical representation of the non-ground LiDAR measurements from the ground segmentation module is converted to 3D cylindrical representation by vertical distribution of measurements. The 2D grid representation is not effective for clustering in cluttered urban scenarios where elevated structures exist. The 3D cylindrical representation allows the clustering of objects that exist under elevated structures, such as streetlights, traffic signals, overhead bridges, and trees. Furthermore, compact representation of data enables efficient computation of clusters.

In 3D grid representation, each cell of the 2D cylindrical grid is further divided into levels based on the vertical height of LiDAR measurements, as shown in Figure 3. For clustering, each cell with measurements is selected as an index and all neighbor cells are traversed to inspect for a threshold number of measurements. In this fashion, the region for each cluster grows with a unique ID. To address the time complexity, the region of interest is selected based on the effective clustering range of LiDAR measurements. The measurements of LiDARs, for example, with fewer channels get sparse at distances, and the actual shape of the object cannot be identified; thus, clustering of points beyond a reliable range only increases the computation and outliers. With these clustering limits imposed, the clustered point cloud is effectively obtained and is treated with the cluster dimension filter. The dimension filter identifies the clusters pertaining to the potentially trackable objects, considering the size, position, and number of enclosed LiDAR measurements. The clusters that are suitable for tracking are forwarded to the bounding box fitting module.

#### 4.1.3. Box Fitting

The LiDAR measurements experience occlusions, and obtaining the exact 3D shape object is inherently impossible. To estimate the actual shape of the clustered object, box-fitting techniques are devised. In this work, considering the computational limitations and real-time requirements, a feature-based method is deployed [36]. The technique represents the cluster in a minimum rectangular shape in 2D top view, then performs L-shape point cloud fitting as shown in Figure 3. Initially, the farthest pair of points is searched for that exists near a threshold boundary of the minimum rectangle boundary, based on the location of the cluster in the spatial space. The pair of points is used to form a line and a farthest point orthogonal to the line is searched. The three points represent the three corners of the 2D area of the clustered object; the height of the cluster is directly used to define the third dimension of the bounding box. The three dimensions of the estimated bounding box are then used to evaluate the centroid and yaw angle of the cluster relative to the LiDAR sensor. The actual dimensions and centroid of the clustered object at this stage has the effect of occlusions; therefore, the tracker module maintains the history of estimated dimensions of the tracked cluster and performs corrections temporally if required.

#### 4.1.4. Object Tracker

The urban cluttered environment perceived by a LiDAR sensor is affected by numerous uncertainties. Cluttered clusters of trackable objects make data association challenging, whereas dynamic objects tend to follow such motion patterns that further increase the uncertainties. To tackle these uncertainties, an IMM–UKF–JPDAF based tracker is used [36]. The Joint Probabilistic Data Association Filter (JPDAF) based approach is utilized for data association with an assumption of Gaussian distribution to address the uncertainties due to clutter. Furthermore, the Interacting Multiple Model (IMM) approach is adopted for the state estimation of objects to deal with the uncertainties due to motion. The non-linearities of the motion models are accommodated by an Unscented Kalman Filter (UKF) through a Gaussian process. The IMM–UKF–JPDAF based approach effectively solves the recursive state estimation and mode probabilities of object clusters, which is described by a non-linear jump Markov system.

The execution times of the tracker module mainly rely on the number of maintained tracks. Furthermore, JPDAF based approaches tend to face a combinatorial explosion problem, which if left untreated, requires exceptional computational resource, as a hypothesis of all possible combinations of tracks and measurements are made. To tackle this shortcoming, a clustering-based scheme is utilized to limit the association pairs of tracks and measurements to only the gated measurements instead of all possible combinations. The tracking block shown in Figure 3 shows a table of tracks T and measurements M in rows and columns, respectively. The track T2 gets gated measurements of M1 and M4 forming an association cluster of two measurements and a single track. Similarly, tracks T3 and T4 share the measurement M3 forming an association cluster of two tracks and a single measurement. Thus, the JPDA problem is reduced to a set of smaller problems that can be solved efficiently. Moreover, an efficient track management mechanism is set in place that prunes out the tracks pertaining to the inconsistent measurements, resulting in a limited number of tracks to maintain. An additional condition of a tracked object being classified by the visual object detector, reducing the number of tracks further, results in decreased computational load. The prime objective of this module is to estimate the tracking parameters of the potentially trackable objects, such as, pose, velocity, and dimensions.

### 4.2. Camera-LiDAR Fusion

The fusion approach adopted in this work is regarded as late fusion, where tracked clusters are temporally classified instead of frame-wise classification [36]. This is carried out by two components, class association and class management. The class association component assigns a visually detected object’s class to the tracked object’s clusters, whereas the class management component assesses the class assignment history to select a class for the tracked object cluster.

To perform the class association, eight corners of the 3D bounding box of the tracked object clusters are projected on the 2D image plane using camera-LiDAR extrinsic parameters. The minimum and maximum pairs of x and y coordinates from the set of 2D projected points forms a 2D bounding box in the image frame representing the 3D tracked object. The Munkres association strategy is applied on the 2D centroids of the visual object detector and 2D bounding box representing the tracked object in the 3D space. This association is maintained by a class association vector that maintains the history of class associations and provides a certainty of classes assigned to the tracked object over time.

Let $T^{k}$ and $D^{k}$ be the sets representing tracks and visually detected objects, respectively, at time step *k*. The centroids of tracked clusters $o_{i}^{k}$ are projected onto the image frame of the corresponding time step, resulting in a 2D pixel location in an image ${\bar{o}}_{i}^{k}$. Similarly, the centroids of visually detected objects are calculated using bounding box dimensions, represented by $m_{j}^{k}$. The 2D centroids of both sources are utilized to populate the Euclidean distance cost matrix $E^{k}=\left[c_{\mathrm{ij}}^{k}\right]$, where i={1, 2, …, T} and j={1, 2, …, D}. The cost matrix

(1) $$c_{\mathrm{ij}}^{k}=\{\begin{array}{cc} d(\bar{o_{i}^{k}}{,m}_{j}^{k}) & \mathrm{if}\;\mathrm{IOU}\left(\bar{t_{i}^{k}}{,d}_{j}^{k}\right)\;>\;0.3\\ 1000 & \mathrm{otherwise} \end{array}$$

is, however, constrained by the criterion that at least 30% of overlap must exist among the corresponding 2D bounding boxes from the two sources. Following the Munkres association, the algorithm for optimized minimum cost is performed and a set of index pairs Υ relating to the associated tracks $t_{i}^{k}$ and visual detections $d_{j}^{k}$ is acquired. Using the set Υ class association matrix ${\hat{E}}^{k}=\left[{\hat{c}}_{\mathrm{ij}}^{k}\right]$, it can be formulated such that,

(2) $${\hat{c}}_{\mathrm{ij}}^{k}=\{\begin{array}{cc} v_{j} & \mathrm{if}<i,j>\;\subset \;\Upsilon \\ 0 & \mathrm{otherwise} \end{array},$$

where v  represents the association of the class of visually detected objects and the dimension index of class association vector $A_{v\;}^{i}{=(a}_{1}^{i}{,\;\ldots a}_{n}^{i})$. The matrix ${\hat{E}}^{k}$ finally updates the class association vector $A^{i}$ and increments the associated class dimension,

(3) $${\hat{c}}_{\mathrm{ij}}^{k}=\{\begin{array}{cc} a_{{\hat{c}}_{\mathrm{ij}}}^{i} & \mathrm{if}\;{\hat{c}}_{\mathrm{ij}}^{k}=0\\ a_{{\hat{c}}_{\mathrm{ij}}}^{i}+1 & \mathrm{otherwise} \end{array}.$$

The class association vector $A^{i}$, along with the age of track $t_{\mathrm{age}}$ is exploited to compute the class certainty $P_{c}^{i}$ of tracked objects and the ratio $P_{o}^{i}$ of the object, that reasons the tracked object to be valid.

(4) $$P_{c}^{i}=\frac{\max\left(a_{v}\right)}{t_{\mathrm{age}}}$$

(5) $$P_{o}^{i}=\frac{\left({\;t}_{\mathrm{age}}-\sum_{v=1}^{n}a_{v}^{i}\right)}{t_{\mathrm{age}}}$$

**Figure 4:**
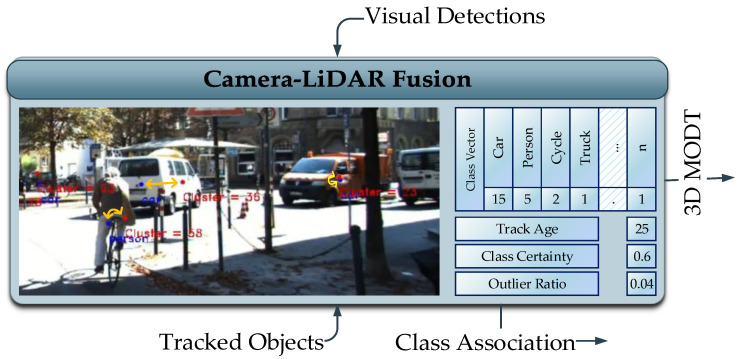
Camera-LiDAR Fusion Module.

The camera-LiDAR fusion thread operates along the LiDAR based MODT and provides the class association certainty to the tracker. In Figure 4, visual classification of tracked clusters is demonstrated. The blue and red dots in the image represent the centroids of visually detected objects and centroids of tracked LiDAR clusters projected on to the image, respectively. The qualified associations update the class association vector of a mature track as demonstrated in Figure 4. The class association vector represents the classes assigned to the tracked cluster, including no associations counting temporally in terms of frames. Together with the tracking age, class certainty of 60% is evaluated for the tracked object being a “car”.

### 4.3. Dynamic Mask Generator

**Figure 5:**
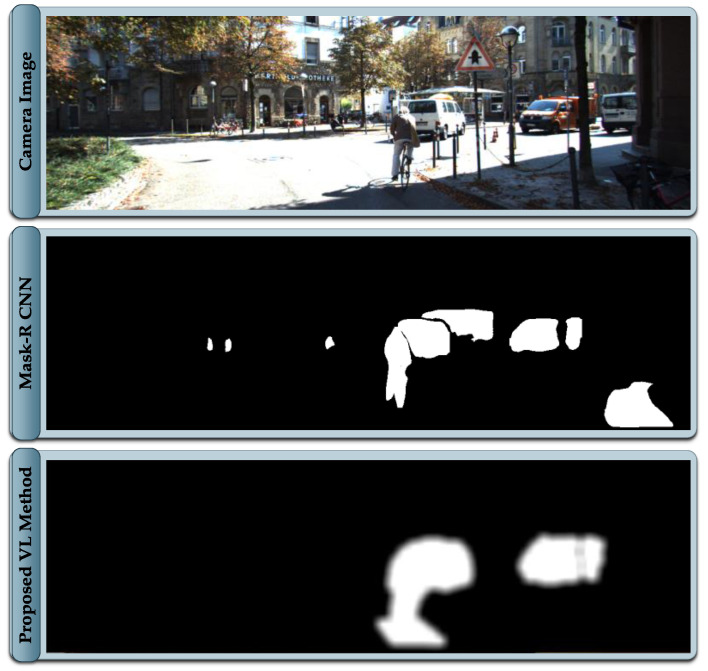
Visual-LiDAR based Mask generated in comparison with Mask R CNN.

The mask generator module takes the LiDAR point cloud cluster and projects it onto the image frame. The visual features generally exist on the edges and corners of the surfaces, and thus, the mask needs to slightly exceed the exact contour of the detected object. To handle the sparsity of the LiDAR measurements, a 2D Gaussian blur kernel is formulated,

(6) $$G(x,\;y)=\frac{1}{{2\pi \sigma }^{2}}e^{\frac{x^{2}{+y}^{2}}{{2\sigma }^{2}}},$$

where x and y are the coordinates of the projected LiDAR measurement pertaining to the cluster of the dynamic object. The standard deviation σ of the blur is set based on LiDAR and image resolution. For lower ratios of image to LiDAR resolution and larger coverage of LiDAR measurements on the image, lower values of σ are sufficient. The dynamic mask generated from the proposed framework in contrast to the mask generated from Mask R-CNN is presented in Figure 5. In the literature, the reported inference time of Mask R-CNN using standard GPU fails to meet the real-time requirements, whereas the proposed framework provides the comparable dynamic masks well within the sensor sampling time, allowing the entire framework to operate within 100 ms.

### 4.4. Object Pose Transformer

The primary function of the object pose transformer is to transform the pose and tracking information of objects to the SLAM frame of reference. The module receives the localization information from the SLAM module and tracking information from the 3D MODT module. The object tracking is performed in the vehicle frame, where static–dynamic classification of tracked objects cannot be effectively made without the ego-motion information. For effective utilization of this information, tracking information is transformed into a SLAM frame of reference, where realistic information of the tracked objects can be realized. 

### 4.5. Dynamic SLAM

**Figure 6:**
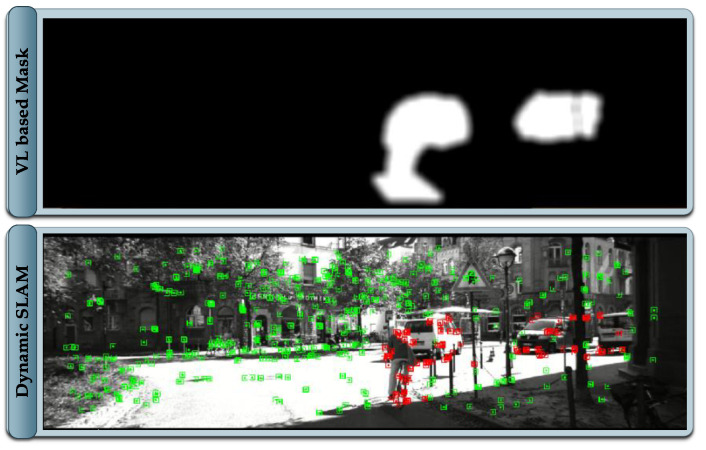
Dynamic SLAM with Visual-LiDAR based dynamic object mask.

The core of the proposed framework is an ORB-SLAM2 that gets the capability of making an informed selection of visual features. The module takes images together with the generated mask image as an input. The extracted visual features are labeled as static or dynamic based on the provided mask. The features pertaining to the static part of the scene are validated, whereas features pertaining to dynamic objects in any state are filtered out, as demonstrated in Figure 6. The focus in this implementation is to demonstrate an effective alternative solution to the computationally expensive semantic segmentation algorithms like Mask R CNN [3], widely utilized in the related literature. The proposed framework generates a comparable dynamic mask in real-time without excessive computational resource requirement.

The visual features generally exist on the corners and sharp edges of the scene, thus extracting a perfect pixel-level annotation and sharp contours of the dynamic objects are not desired, as features located on the boundary of dynamic objects are dubious. The masks generated in the proposed framework are based on sparse LiDAR data that is treated by a 2D Gaussian blur that ensures that features on the boundary regions of dynamic objects are filtered. Consequently, the generated map is free of information pertaining to dynamic objects, although the tracker module allows identification of the dynamic objects that are currently static such as parked cars and using this information within the SLAM process of pose estimation. This implementation is intentionally left out for future work, as the focus is to demonstrate that a comparable dynamic object’s mask to the conventional mask generation approaches can be efficiently achieved. In this work, LiDAR data are only used for spatial object tracking and exploitation of a classified point cloud for dynamic mask generation.

## 5. Evaluation and Comparison

To evaluate and analyze the effect of the proposed Dynamic SLAM framework, a comparison is made between basic ORB-SLAM2 [26] and the proposed MODT based dynamic SLAM framework. Conventionally, KITTI Datasets [27] are used for fair evaluation against the provided ground truths over well-established evaluation metrics. However, the datasets provided for evaluating SLAM algorithms lack in the presence of dynamic objects, mainly because of static world assumption. For this reason, the datasets provided for object tracking are used with the provided ego-motion data as the ground truth. The true potential of the framework can be fairly tested in the absence of no loop closures, allowing the measurement of maximum drift. Since the datasets are targeted to test object detection and tracking, dynamic objects exist in almost every sequence in abundance. 

To perform a detailed and standard analysis, the KITTI Odometry criterion [27] is followed. The Absolute Trajectory Error (ATE) measures the root-mean-square error between predicted camera poses [x, y, z] and ground truth. Relative Pose Error (RPE) measures frame-to-frame relative pose error, since the provided ground truth data are in the GPS/IMU frame, and the trajectories generated by SLAM are in camara coordinates. The generated trajectory is aligned using 7DoF optimization to the ground truth associated poses during evaluation by minimizing the ATE [37]. Table 1. The estimated trajectories evaluation and comparison on KITTI Tracking Datasets

The results of comparison are presented in Table 1. The variation in the scores suggests the presence of dynamic objects. The better performing metrics are presented in bold text. The sequences with dynamic objects having less effect on the SLAM produce near similar metric results. In these sequences the dynamic objects’ presence does not cover the entire view for consecutive time frames. In some sequences, the dynamic objects are static, like parked cars, the conventional SLAM takes use of the features belonging to the corresponding regions to produce better results. However, this information becomes the part of the generated maps.

**Figure 7:**
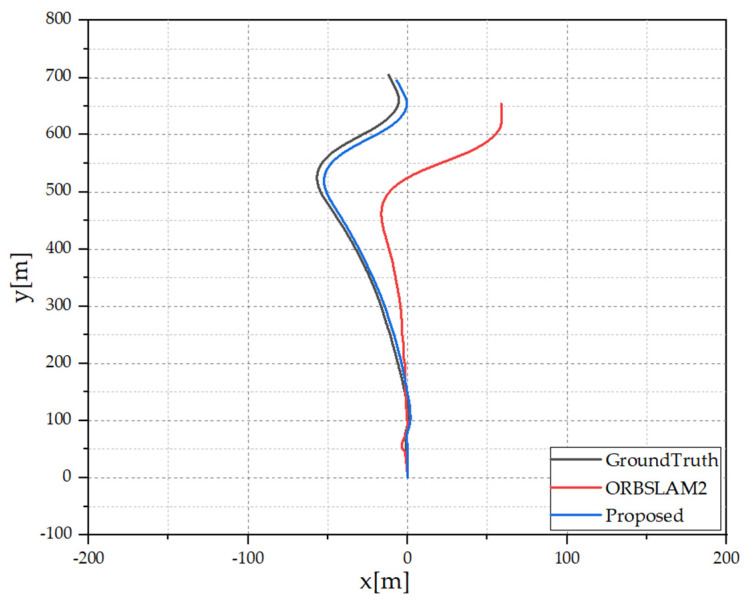
Estimated trajectories of sequence 20 from KITTI Tracking Dataset.

The sequences with dynamic objects are in a motion similar in pattern to the ego-vehicle, such as in the roadway conditions, influencing SLAM the most. The metric scores of sequences 9, 19, and 20 support this argument, where ORB-SLAM2 [26] suffers a lot due to the presence of dynamic objects, and drift creeps into the estimated trajectory. In contrast, the proposed framework efficiently filters the visual features pertaining to the dynamic object regions. Thus, the estimated trajectory is more accurate. The plots of trajectories generated by ORB-SLAM2 [26] and the proposed framework of sequence 20 are presented in Figure 7. The drift in both plots suggest that the informed selection of features can estimate better trajectories, specifically in the scenario where dynamic objects tend to follow a similar motion pattern.

To view the performance of proposed framework in comparison to the state-of-the-art dynamic SLAM frameworks, the metric scores against KITT Raw DATASET [28] sequences are evaluated and presented in Table 2. The evaluation scores of ORB-SLAM2 [26] are reproduced using the similar evaluation tool and computational platform. However, the evaluation scores of related dynamic SLAM frameworks on the same dataset sequences are reported as a reference from the work in [25]. Best mean metric scores suggest that the proposed dynamic SLAM framework can perform at par to the conventional approaches that rely on data driven semantic segmentation approaches to identify dynamic objects in the scene.

The mean overall scores reflect the promising performance of the proposed algorithm but the individual sequences of 0009, 0014, and 0004 show slightly poor results. The similarity in these sequences is the abundance of dynamic objects being static. ORB-SLAM2 making use of the whole visible region can produce better pose estimation, whereas the dynamic SLAM ignoring the dynamic regions tends to degrade the performance in such scenarios. However, this can be seen as an advantage in terms of SLAM process, where the generated map remains free of dynamic entities, enabling better loop closure and re-localization capabilities. The sequence 0013 is a relatively short time sequence with limited dynamic regions and plentiful visual features; thus, dynamic SLAM tends to produce similar metric scores. Sequence 0047 being a highway scenario with a large trajectory demonstrates the effectiveness of the proposed dynamic SLAM. An interesting find in the reported scores is that the tightly coupled approaches perform more poorly than the loosely coupled approaches. ClusterVO [21], and DynaSLAM II [25] having a tightly coupled approach tend to have lower accuracies on average. On the other hand, DynaSLAM [29] which follows a dynamic masking approach like the proposed framework manages to acquire better metrics in comparison. ClusterSLAM [24] largely relies on the initial camera pose estimations, which further supports the argument that loosely coupled approaches with effective use of redundant information can guarantee better accuracies.

## 6. Conclusions

In this work, a visual-LiDAR based 3D MODT is integrated with SLAM that caters to the challenges pertaining to the dynamic world. The proposed framework considering the constrained computational resources and real-time requirements performs a temporal classification of tracked objects. An efficient IMM–UKF–JPDAF based tracker spatially tracks the objects while maintaining the class association history, to address the real-time limitations and shortcomings of object detection. The classified LiDAR point cloud is effectively utilized to produce a dynamic object mask, capable of replicating the state-of-the-art semantic segmentation approaches. SLAM exploits the dynamic mask provided by the MODT for informed selection of visual features for tracking and mapping tasks to realize a dynamic SLAM.

The proposed framework is tested on the KITTI Datasets [28] and evaluated against the established metrics. The results and comparison with the related works suggest that the proposed approach is an effective solution for the dynamic SLAM. The key contribution in this work is the efficient generation of dynamic object mask from the Visual-LiDAR based MODT in real-time without the need of exceptional computational resources. Furthermore, a loosely coupled approach for 3D MODT and SLAM is proposed that can exploit the redundant information with a minimalistic interdependence.

In the future, it is intended to incorporate the further static–dynamic classification of characteristically dynamic objects supported by the MODT information. Furthermore, information pertaining to static regions of the scene can be utilized in semantic representation on the maps. Moreover, the tracking and semantic information of the dynamic objects in the environment is at the disposal of SLAM framework, which can be exploited at several stages of SLAM to attain better accuracies and generation of semantic maps.

## Figures and Tables

**Table 1:** The estimated trajectories evaluation and comparison on KITTI Tracking Datasets.

seq	ORB-SLAM2 [26]			Proposed Framework		
	ATE (m)	RPE (m)	RPE (deg)	ATE (m)	RPE (m)	RPE (deg)
00	0.663	0.63	1.345	**0.663**	**0.63**	**1.345**
01	**1.362**	**1.062**	**0.472**	1.704	1.066	0.474
02	**0.127**	**0.704**	0.139	0.148	0.705	**0.139**
03	0.254	**1.699**	0.245	**0.245**	1.703	**0.244**
04	1.73	**1.803**	0.589	**1.275**	1.805	**0.583**
05	0.58	**1.717**	0.117	**0.557**	1.719	**0.116**
06	**0.175**	0.263	**0.481**	0.366	0.254	0.492
07	6.122	0.914	1.107	**3.784**	**0.906**	**1.042**
08	**1.346**	**2.092**	**0.259**	1.369	2.093	0.267
09	8.08	1.289	0.563	**1.677**	**1.26**	**0.521**
10	**0.472**	2.02	0.269	0.55	**2.02**	**0.267**
11	1.324	0.826	0.171	**0.95**	**0.824**	**0.17**
12	0.01	0.003	0.006	**0.01**	**0.002**	**0.005**
13	**0.451**	0.818	**0.383**	0.462	0.818	0.384
14	**0.423**	0.642	**1.436**	0.505	0.634	1.456
15	0.581	0.282	0.04	**0.31**	**0.274**	**0.039**
16	0.023	0.008	0.006	**0.022**	**0.008**	**0.006**
17	0.037	0.006	**0.008**	**0.037**	0.004	0.009
18	**0.866**	**1.088**	**0.118**	1.032	1.096	0.119
19	3.742	0.324	0.26	**1.835**	**0.315**	**0.24**
20	7.891	1.227	0.261	**0.634**	**1.219**	**0.25**
mean	1.812	0.970	0.413	**0.906**	**0.967**	**0.408**

Lower errors are written in bold.

**Table 2:** The estimated trajectories evaluation and comparison on KITTI Raw DATASET.

seq	ORB-SLAM2 [26]			DynaSLAM [29]			ClusterSLAM [24]			ClusterVO [21]			DynaSLAMII [25]			Proposed Framework		
	ATE [m]	RPE [m]	RPE [deg]	ATE [m]	RPE [m]	RPE [deg]	ATE [m]	RPE [m]	RPE [deg]	ATE [m]	RPE [m]	RPE [deg]	ATE [m]	RPE [m]	RPE [deg]	ATE [m]	RPE [m]	RPE [deg]
0009	1.36	1.06	0.47	0.81	1.80	0.57	0.92	2.34	1.72	**0.79**	2.98	1.72	0.85	1.87	0.57	1.70	**1.06**	**0.47**
0013	0.25	1.69	0.24	0.30	0.99	0.57	2.12	5.50	4.01	0.26	1.16	0.57	0.29	**0.93**	**0.00**	**0.24**	1.70	0.24
0014	1.73	1.80	0.58	0.60	1.62	**0.57**	0.81	2.24	1.72	**0.48**	**1.04**	**0.57**	**0.48**	1.35	**0.57**	1.27	1.80	0.58
0004	0.86	**1.08**	**0.12**	0.56	1.36	0.57	1.12	2.78	1.15	**0.40**	1.77	1.15	0.64	1.41	0.57	1.03	1.09	**0.12**
0047	7.89	1.22	0.26	2.87	5.95	1.15	10.2	8.94	3.44	4.78	6.54	2.86	3.03	6.85	1.15	**0.63**	**1.21**	**0.25**
mean	2.42	1.37	0.33	1.02	2.34	0.68	3.03	4.36	2.40	1.34	2.69	1.37	1.05	2.48	0.57	**0.97**	**1.37**	**0.33**

Better and similar scores are written in bold.

## Data Availability

Not applicable.
